# Bridging the Gap: Parental Supervision as a Mediator Between Home Environment and Unintentional Injuries in Children Under 3 Years

**DOI:** 10.3389/ijph.2025.1606726

**Published:** 2025-02-20

**Authors:** Jianlin Ji, Hanlin Yang, Chengxi Zeng, Ouyao Chen, Qunfeng Lu

**Affiliations:** ^1^ School of Nursing, Shanghai Jiao Tong University, Shanghai, China; ^2^ Shanghai Sixth People’s Hospital, Shanghai Jiao Tong University, Shanghai, China

**Keywords:** health promotion, children, unintentional home injury, parental supervision, home environment

## Abstract

**Objectives:**

Unintentional home injuries pose a serious risk to children under 3 years. While parental supervision and home environment are key factors influencing these injuries, few studies have explored the correlation between them. This study aimed to examine the relationship between home environment, parental supervision, and unintentional home injuries, and to investigate whether parental supervision mediates this relationship.

**Methods:**

This study, conducted in Shanghai, China from June to August in 2023, using a convenience sample. Paper questionnaires were distributed to 600 parents of children, assessing unintentional injuries, parent supervision (using parent supervision attributes profile questionnaire), in-home environmental risk scale and demographic variables. A bootstrap test was applied to assess the mediating role of parental supervision.

**Results:**

Both in-home environment risks and parental supervision were significantly related to unintentional home injuries in children under 3. Moreover, parental supervision was found to partially mediate the relationship between environmental risks and injuries, explaining 14.2% of the total effect.

**Conclusion:**

Children under 3 were highly vulnerable to unintentional home injuries. Enhancing parental supervision may reduce the impact of environmental risks on injury occurrence. These findings offer practical guidance for health practitioners, underscoring the value of community-based interventions and tailored educational programs for injury prevention. Future research should explore intervention effectiveness and long-term outcomes.

## Introduction

Unintentional injuries (UI) in childhood represent a significant global public health concern, being the leading cause of mortality in children. According to the Global Burden of Diseases report, more than 200,000 child deaths occur annually as a result of UIs [1], with the majority of these injuries taking place within the home [2]. Unintentional home injuries (UHI) substantially contribute to the global disease burden. In China, the incidence rate of UHI was 65.5%, with mortality rates exceeding those in developed countries by 3–11 times [3]. In 2019, UHIs accounted for 12.49% of all child deaths in China, with nearly 1.9 million Disability-Adjusted Life Years lost among children and adolescents [4]. Thus, great attention should be paid to the risk factors of UHI so as to develop strategies to reduce the incidence of UHI.

Age is an important predictor of UHI, with younger children at greater risk of UHI [5]. Specifically, children aged 0–3 years are the main age group for UHI compared to other age group [6]. Given the heightened vulnerability of this population, it is essential to examine UHI within this age group. Furthermore, the home environment is subject to frequent changes, and parenting practices play a pivotal role in child safety. Due to their physical and cognitive immaturity, children under the age of 3 are highly dependent on their caregivers for protection from injury [7, 8]. As primary caregivers, parents have a critical role in preventing childhood injuries [7]. According to the Haddon model, which provides a framework for understanding UI, key risk factors include both the physical and social environments, as well as interpersonal interactions [9]. So, we focused on the home environment and parental supervision as key factors influencing UHI.

The home environment encompasses both indoor areas and associated outdoor spaces, such as balconies, rooftops, and gardens [10]. Most studies have identified home environment as a major factor to UHI [6, 10–12]. Each additional injury hazard observed in the home was associated with a 22% increase in the odds of injury [13]. Many home hazards are often overlooked yet can significantly increase the risk of injury. For example, 41% of homes had unsafe water temperatures [14] and only 10% of families with young children securely store toxic substances [15]. Additionally, 97% of families leave prescription medications within reach of children [16]. Therefore, exploring their home surroundings is crucial to children’s UHI. Furthermore, the home environment has been shown to correlate with parental supervision [12].

Parental supervision is defined as “the interaction between attentive behaviors and physical proximity over time” [17]. Inadequate supervision of young children is widely recognized as a significant contributing factor to UHI. Children under five who died from UIs were 3.3 times more likely to be unattended than children who were alive [17]. In addition, Schnitzer et al [18] demonstrated a significant association between lower levels of parental supervision and an increased risk of injury, which aligns with the findings of Yang et al [19]. However, research also shows that children may still face injury risks even when closely supervised [20]. It is therefore crucial to explore the complex interactions between parental supervision and UHI.

Despite growing research in this area, few studies have speciafically examined the correlation regarding home environment, parental supervision and UHI, particularly regarding the potential mediating role of supervision. Morrongiello et al. [21] highlighted the need for future research to assess how environmental characteristics interact with caregiver behaviors to influence children’s risk of UI. Consequently, this study aims to describe the current state of UHI among children and investigate the relationships between home environment, parental supervision, and UHI.

## Methods

### Aim

This study aims to explore in-home environmental risks and parent supervision in relation to UHI and the mediating role of parent supervision between in-home environmental risks and parent supervision among younger children (0–3 years).

### Design

A descriptive, cross-sectional design was applied using a convenience sample.

### Participants

The study included fathers or mothers aged 18 or older who accompanied their children aged 0–3 to a tertiary children’s hospital in Shanghai from June to August 2023. Parents whose children have severe organic disease or were unwilling to sign an informed consent form were excluded.

The hospital was chosen as the site for investigating child injuries because it provides immediate access to injured children and their families, allowing researchers to gather detailed information on the causes, injury specifics, and parental supervision at the time of the incident. As a centralized hub, the hospital offers diverse cases, ensuring a more representative sample. Additionally, parents in this setting are often more open to sharing incident details and seeking guidance, enabling real-time data collection, reduced recall bias, and improved data accuracy.

### Measurement Instruments

#### The Demographic Questionnaire

Demographic information assessed the characteristics children (age, gender, only child, residence et al) and their families (education level of parents, living with grandparents et al.).

#### Measurements of Unintentional Injury

According to the International Classification of Diseases, 10th edition [22], and the characteristics of children aged 0–3 years, the unintentional injuries criteria were established: (a) children need to visit a medical unit and have a specific type of injury diagnosed, or (b) children need emergency treatment or care by family members, or (c) children have restricted activity more than half a day.

Before asking whether the child had suffered an UHI in the last 12 months, we described what an UHI is. If the child had suffered an UHI, parents answered the number of times and selected the type of unintentional injury. (Question-1 Have your children suffered an unintentional injury in the last 12 months; Question-2- How many unintentional injuries have your children had in the last 12 months; Question-3 Please fill in the type and number of injuries your child has had in the last 12 months) The responses were converted into scores on a 5-point Likert scale, primarily based on the frequency indicated in Question 2.

### Parent Supervision Attributes Profile Questionnaire

Parent supervision attributes profile questionnaire (PSAPQ) is a 29-items tool designed to assess various aspects of parental supervision [23]. It covers four dimensions: protectiveness with 9 items, supervision beliefs with 9 items, tolerance for children’s risk-taking with 8 items, and belief in fate with 3 items. Items were rated on a 5-point Likert scale ranging from 1 (never) to 5 (all of the time). The total score varies from 29 to 145 and higher total scores indicated parents had more engagement with supervision for their children. The PSAPQ has good reliability and validity in the Chinese population [19]. In the current study, Cronbach’s alpha for the scale was 0.847.

### In-Home Environmental Risks Scale

The In-home Environmental Risks Scale, derived from the Knowledge, Attitudes, and Behavior in Unintentional Injuries Questionnaire developed by Ma et al [7], is used to assess the safety of the home environment. It consists of 27 items focusing on various aspects of potential environmental risks within the home, such as hazards that could contribute to unintentional injuries. Each item is rated on a 5-point Likert scale (1 = never, 5 = always), with higher scores indicating a safer home environment as perceived by the parents. In this study, the scale demonstrated strong internal reliability, with a Cronbach’s alpha of 0.876.

### Data Collection

Prior to initiating data collection, a research team was formed, and primary investigators provided standardized training to all team members. This training encompassed the procedures for completing the questionnaire, study objectives, inclusion and exclusion criteria for participant selection, and key considerations to ensure accurate and consistent questionnaire completion.

Data for the study was collected with paper surveys that were posted in June to August in 2023. Before participation, each potential respondent was informed about the study’s purpose, and informed consent was obtained. Participants were also notified of their right to withdraw at any stage of the study. Upon completion of each questionnaire, researchers reviewed responses for completeness and requested clarification on any missing items, ensuring the accuracy and integrity of the data. For parents unable to complete the questionnaire independently due to childcare responsibilities, one-on-one, face-to-face interviews were conducted. Interviewers adhered to a structured, standardized protocol to guide participants through each question. A total of 600 questionnaires were received. After screening the incomplete and visibly unqualified questionnaires, 594 were included in the statistical analysis, with the response rate of 99%.

### Data Analysis

After data were collected, the statistical analyses were performed using SPSS version 26 and Mplus 8.3. Descriptive statistics were adopted to analyze the demographic characteristics of all participants, means and standard deviation (SD) of three variables. Pearson’s correlation coefficients were calculated to test the correlated relationship among three research variables. Multiple linear regression was used to evaluate whether in-home environment and PSAPQ could significantly affect the UHI. In order to obtain the confidence intervals, bootstrapping was performed for each of the models with 1,000 replications. The mediation effect is significant (p < 0.05) if the confidence interval constructed does not include 0.

## Results

### Characteristics of UHI

The proportion of children who experienced UHI was 53.2% (n = 316). Additionally, 145 children were injured once, 82 children were injured twice, and 61 children were injured three, 28 children were injured forth or more. The prevalence rate of recurrent UIs was 47.3%. The most frequently reported type of UHI was falls, followed by cuts/stabs and burns.

### Univariable Analysis Based on the UHI History

There were 594 participants in this study, ageing from 0 to 3 years old, with an average age of 2.01 ± 0.92 years old. The average scores for the In-home Environmental Risk and PSAPQ were 107.91 ± 16.90 and 105.20 ± 13.88, respectively. Specifically, children with UHI had significantly lower scores on both the PSAPQ and the In-home Environmental Risk compared to those without injury, indicating that higher scores in these measures are associated with greater child safety. A significant difference was revealed between the children’s age. The participants’ demographic characteristics are outlined in Table 1.

**TABLE 1:** The sociodemographic characteristics of participants (Shanghai, China. 2023).

Variables	Total	Without IUIs history	With IUIs history	X^2^/F	P
Age	2.01 ± 0.92	1.79 ± 1.0	2.19 ± 0.79	24.66	<0.001
Sex				0.140	0.80
Male	346 (58.2)	160 (46.2)	186 (53.8)		
Female	248 (41.8)	118 (47.6)	130 (52.4)		
Premature				0.39	0.56
Yes	51 (8.6)	26 (51)	25 (49)		
No	543 (91.4)	252 (46.4)	291 (53.6)		
Residence				0.003	1.0
City	508 (85.5)	238 (46.9)	270 (53.1)		
Town	86 (14.5)	40 (46.5)	46 (53.5)		
Only child				0.031	0.928
Yes	421 (70.9)	198 (47)	223 (53)		
No	173 (29.1)	80 (46.2)	93 (29.4)		
Education level of mother				4.94	0.083
Technical secondary school and below	93 (15.7)	36 (38.7)	57 (61.3)		
Junior college	124 (20.9)	53 (42.7)	71 (57.3)		
Bachelor’s degree or above	377 (63.5)	189 (50.1)	188 (49.9)		
Education level of father				0.74	0.70
Technical secondary school and below	88 (14.8)	39 (44.3)	49 (55.7)		
Junior college	130 (21.9)	58 (44.6)	72 (55.4)		
Bachelor’s degree or above	376 (63.3)	181 (48.1)	195 (51.9)		
Home environment	107.91 ± 16.90	113.74 ± 15.58	102.78 ± 16.36	4.94	<0.001
Protectiveness	34.90 ± 5.37	35.53 ± 5.01	34.36 ± 5.63	4.22	0.008
Tolerance for children’s risk taking	29.66 ± 5.31	30.09 ± 5.47	29.28 ± 5.16	2.25	0.062
Belief in fate	6.40 ± 2.89	6.49 ± 3.08	6.33 ± 2.71	2.32	0.49
Supervision beliefs	34.31 ± 5.60	35.24 ± 5.49	33.50 ± 5.57	0.01	<0.001
PSAPQ	105.20 ± 13.88	107.35 ± 14.0	103.31 ± 13,51	1.14	<0.001

Abbreviation: PSAPQ: parent supervision attributes profile questionnaire.

### The Correlated Relationship Among UHI, PSAPQ, and In-Home Environmental Risks

The results of correlational analyses among UHI, PSAPQ, and in-home environmental risks were shown in Table 2. UHI were positively associated with age (r = 0.166) and negatively associated with in-home environmental risks (r = −0.261) and PSAPQ (r = −0.230).

**TABLE 2:** Correlations between the study variables (Shanghai, China. 2023).

Variable	1	2	3	4	5	6	7	8
1. Age	-	0.166^**^	−0.095^*^	−0.015	0.066	−0.057	−0.026	0.003
2. UHI	0.166^**^	-	−0.261^**^	−0.184^**^	−0.131^**^	−0.032	−0.231^**^	−0.230^**^
3. In-home environmental risks	−0.095^*^	−0.261^**^	-	0.217^**^	0.100^*^	−0.006	0.181^**^	0.197^**^
4. Protectiveness	−0.015	−0.184^**^	0.217^**^	-	0.342^**^	0.045	0.641^**^	0.775^**^
5. Tolerance for children’s risk taking	0.066	−0.131^**^	0.100^*^	0.342^**^	-	0.117^**^	0.568^**^	0.757^**^
6. Belief in fate	−0.057	−0.032	−0.006	0.045	0.117^**^	-	0.010	0.265^**^
7. Supervision beliefs	−0.026	−0.231^**^	0.181^**^	0.641^**^	0.568^**^	0.010	-	0.863^**^
8. PSAPQ	0.003	−0.230^**^	0.197^**^	0.775^**^	0.757^**^	0.265^**^	0.863^**^	-

Abbreviation: PSAPQ, parent supervision attributes profile questionnaire.

*: P value < 0.05 **: P value < 0.01.

### Multiple Linear Regression Analysis of UHI

A multiple linear regression model was constructed using UHI as the dependent variable. Age, in-home environmental risks and PSAPQ were used as the independent variables. In-home environmental risks (β = −0.189, P < 0.01) and PSAPQ (β = −0.209, P < 0.01) were negatively correlated with UHI, while age was positively correlated with UHI. Detailed results of the regression analysis are shown in Table 3.

**TABLE 3:** The multiple linear regression analysis (Shanghai, China. 2023).

Variables	B	SE	Beta	t	p
Age	0.185	0.049	0.146	3.779	0.001
PSAPQ	−0.015	0.003	−0.209	−5.295	<0.001
In-home environmental risks	−0.016	0.003	−0.189	−4.797	<0.001

Abbreviation: PSAPQ: parent supervision attributes profile questionnaire.

### The Mediating Effect of PSAPQ Between In-Home Environmental Risks and UHI

As shown in Table 4, in-home environmental risks directly influenced UHI and indirectly affected UHI through PSAPQ. The indirect effect accounted for 14.2% (−0.037/−0.261) of the total effect of in-home environmental risks and UHI.

**TABLE 4:** The results of the mediation analysis (Shanghai, China. 2023).

Path	Estimate	P	BCa 95% CI
Total effect	−0.261	<0.001	−0.025 ∼ −0.012
Direct effect	−0.224	<0.001	−0.022 ∼ −0.010
Home environment-PSAPQ- UHI	−0.037	0.001	−0.004 ∼ −0.001

Abbreviation: BCa 95% CI: the bias-corrected and accelerated 95% confidence interval, PSAPQ, parent supervision attributes profile questionnaire.

## Discussion

In this study, around half of children had suffered UHI in the previous year, higher than the results in other studies [12, 24]. It also examined the mediating influence of parental supervision on the association between risks present in the home environment and UHI in young children aged 0–3. To our knowledge, this is the first to explore the connection between parental supervision, home environment risks, and UHI. Additionally, the model employed evaluates the intricacies of UHI processes which exist in the real world (such as the interactions between caregiver and environmental traits) when studying children’s risk of injury.

### Age-Related Vulnerabilities: Limited Motor Control and Increased Curiosity Drive Higher UHI Incidence

The incidence of unintentional injuries (UHI) among infants and young children aged 0–3 can reach up to 50% [6], with 53.2% of children in our study experiencing UHI. This high rate likely stems from age-specific physical and cognitive development characteristics. During this period, rapid growth and intense curiosity drive children to explore their surroundings, yet limited motor skills, self-protection awareness, and cognitive abilities leave them vulnerable to injury [25]. Lacking mature motor control and risk awareness, young children are often unable to recognize or avoid dangers effectively [26]. The results of multiple linear regression analysis showed that age plays a crucial role in predicting UHI. Furthermore, compared to children without a history of UHI, we have found that children with an average age of 2.19 ± 0.79 are more prone to suffer from them. This aligns with a previous study conducted in Wenzhou, which showed that children aged between 24 and 47 months were more susceptible to injuries at home [24]. As infants grow, their motor skills at two different stages of motor development (pre-mobile, mobile). However, limited control over these abilities and an underdeveloped sense of risk make them prone to accidents [27]. In addition, the most common injury in our study reported is fall, which is in line with Ma et al [7]. Their relatively large heads and higher center of gravity, however, make them prone to losing balance and falling, often leading to head or other injuries [28]. When developing interventions to reduce UHI, it is crucial to consider the developmental characteristics and common injury types associated with each age group. A thorough analysis of age-specific risk behaviors and their evolution can offer parents valuable insights into the potential hazards children encounter as they explore the home environment. This approach not only enhances the effectiveness of interventions but also serves as an essential resource for parents, particularly first-time parents, helping them to better prevent injuries and establish a safer home environment for their children.

### Home Environment Risks: Overlooked Hazards and the Importance of Parental Awareness

There was a substantial adverse correlation found between home environment risks and UHI in this study. Similarly, a study conducted in Nepal indicated that an increase in home injury hazards led to a rise in child injury incidence [29]. In addition, many potential sources of danger in the home environment can be easily overlooked, such as home structures (slippery floors, stairs/steps, bathtubs/showers) and furnishings (mainly beds, chairs and rugs/carpets/doormats), small objects (e.g., coins, buttons, screws, cotton and nylon containers), medicines, chemical substances, sharp objects (knives, razors, glass and containers) that can lead to UI [11]. However, environmental changes may be effective in reducing home hazards [13] and parental awareness plays an integral role in this process. In particular, parental risk perception is a facilitator of parents’ efforts to prevent UHI [30]. Studies show that strengthening risk perception among families of affected children is essential. Parents more receptive to threat-related information are likelier to take preventive action. Pang et al [31] used visuals showing the child’s parents the whole process from various risk factors, including home environment, to injury to improve parents’ situational awareness of UHI and reduce home environment risk factors accordingly. In addition, community-led, sustained safety education can enhance child safety awareness, improve parental knowledge, and foster a safer environment for children. Feng et al [5] conducted a WeChat-group-based parental health education including making changes to the home environment centered on community-based basic public services, and the results showed that this health education program could decrease the occurrence of IUIs by enhancing the safety consciousness of parents of children under 3 years old to modify children’s surroundings. We recommend a comprehensive, community-based child injury prevention program that includes accident prevention, home safety enhancement, parental safety awareness, and emergency response planning, emphasizing the community’s critical role in UHI prevention. Additionally, given that caregivers may be distracted by children’s needs, engaging visual materials (slides, videos) combined with demonstrations and interactive parent sessions are suggested to maintain focus. Regular family meetings are also encouraged to systematically identify and reduce household hazards, further lowering overall risk levels.

### Parental Supervision as a Protective Factor: Mediating Environmental Risks and UHI

The study results indicate that children with lower levels of parental supervision experience a higher incidence of UHI, consistent with previous research findings [17]. This result could be attributed to the influence of parental supervision on children’s behaviors; by actively supervising, parents play a critical role in mitigating risky behaviors, thereby reducing the likelihood of UHI [32]. This study identified that parental supervision partially mediates the relationship between home environmental risks and UHI. Social Cognitive Theory, which emphasizes the role of environmental factors in shaping behavior through observational learning and self-efficacy [33], supports parental supervision as a critical protective factor. Within this framework, parental supervision functions as both a risk management strategy and a behavioral model for children. By actively monitoring their children’s activities and promptly mitigating hazards, parents create a buffer against environmental risks, reducing the likelihood of injury [30, 33]. Additionally, children who observe their parents’ proactive behaviors gradually internalize these safety practices, developing the ability to recognize and avoid risks independently. Thus, through consistent and engaged supervision, parents not only reduce immediate dangers but also instill safer habits in their children, leading to a meaningful decrease in UHI incidence. However, this finding contrasts with those of Ma et al. [12], who reported that parental supervision did not mediate the relationship between home environmental risks and UHI, suggesting that supervision was not a significant factor in reducing injury risk for children with an average age above 3 years, either directly or indirectly, as shown through structural equation modeling. The potential reason for this discrepancy could be that parental supervision styles vary across different age groups. Children aged 0–3 years tend to be highly mobile and display more risk-taking behaviors, increasing their vulnerability to UHI [17, 28]. Consequently, parents of younger children typically emphasize both the frequency and proximity of supervision. As children grow older, however, parents may underestimate the need for close supervision or may assume that their children can independently manage risks of injury [17, 34]. Morrongiello et al. [35] developed the “Supervising for Home Safety” program, aimed at helping parents decrease unsupervised time, increase direct visual supervision, and extend monitoring even when children are out of sight. Results confirmed that increased supervision through this program effectively reduced injury risk, underscoring the protective role of parental supervision in preventing UHI.

However, there is currently no comprehensive definition of what constitutes adequate supervision. Future research on parental supervision should, therefore, focus on developing a robust conceptual analysis to clarify what defines adequate parental supervision. Additionally, research should prioritize evaluating and enhancing the effectiveness of parental supervision interventions specifically aimed at preventing UHI in children aged 0–3.

### Limitations

Despite some important findings in this study, there are several limitations to the extension of our results to the wider population. Firstly, the cross-sectional design used in the present study cannot establish a causal relationship between home environment risks, parental supervision and UHI. Future research could employ a longitudinal design to explore the causal relationship, particularly between parental supervision and UHI, across different age groups. Additionally, examining the supervisory behaviors of mothers and fathers separately could provide insights into how each parent’s supervision style influences injury risk, thus enabling the development of more targeted and specific strategies to reduce the occurrence of unintentional injuries. Secondly, the study recruited participants from a hospital setting rather than the community, which may not fully represent the general population’s UHI-related characteristics. The choice to recruit from a hospital setting was based on accessibility to a concentrated group of affected families, ensuring a sample with firsthand experience of UHI. However, this may introduce a selection bias, as these families might differ from those in community settings regarding injury risk perception and management behaviors. Third, conducting this study in Shanghai, China, may limit the applicability of our findings to other regions. Shanghai, as a large, urbanized city, presents unique environmental and social factors that may differ from other areas. Studying UHI in Shanghai provides valuable insights into urban injury prevention and parental supervision patterns, which can serve as a reference for other metropolitan areas. Nonetheless, further research across diverse geographic regions in China is needed to better understand the regional variations in parental supervision and UHI risk for children under three.

### Conclusion

This study showed that in-home environment risks and parental supervision affected UHI among children under 3 years, while parental supervision partially mediated the relationship between in-home environment risks and UHI. As the primary environment for young children, the home requires parents to eliminate safety hazards and maintain strong safety awareness. By strengthening supervision, parents play a crucial role in reducing unintentional injuries. However, injury prevention should extend beyond family efforts; community support in education and resources is also essential. Health practitioners should encourage community engagement by establishing support networks that provide families with essential resources and education. Community-based interventions can empower parents to manage risks effectively, offering practical guidance on supervision and environmental safety. Specifically, community programs tailored to children’s age, gender, and seasonal risks are crucial. By focusing on injury prevention knowledge, basic first-aid skills, and health guidance, these programs build a collaborative framework in which families and communities work together to ensure children’s safety.
